# Single image super-resolution based on approximated Heaviside functions and iterative refinement

**DOI:** 10.1371/journal.pone.0182240

**Published:** 2018-01-12

**Authors:** Xin-Yu Wang, Ting-Zhu Huang, Liang-Jian Deng

**Affiliations:** School of Mathematical Sciences/Research Center for Image and Vision Computing, University of Electronic Science and Technology of China, Chengdu, Sichuan, P. R. China; Chongqing University, CHINA

## Abstract

One method of solving the single-image super-resolution problem is to use Heaviside functions. This has been done previously by making a binary classification of image components as “smooth” and “non-smooth”, describing these with approximated Heaviside functions (AHFs), and iteration including *l*_1_ regularization. We now introduce a new method in which the binary classification of image components is extended to different degrees of smoothness and non-smoothness, these components being represented by various classes of AHFs. Taking into account the sparsity of the non-smooth components, their coefficients are *l*_1_ regularized. In addition, to pick up more image details, the new method uses an iterative refinement for the residuals between the original low-resolution input and the downsampled resulting image. Experimental results showed that the new method is superior to the original AHF method and to four other published methods.

## Introduction

Image super-resolution (SR) is to generate or recover high-resolution (HR) images from *one* or *multiple* low-resolution (LR) images. If we generate/recover the HR image from only one LR image, we call it *single-frame* SR. Otherwise, we call it *multiple-frame* SR. The multiple-frame SR methods are available in that multiple LR images are of the same scene with different sub-pixel shifts taken as input. It has direct applications in video SR problems (see [1]). *Single-frame* SR methods are quite popular and challenging when only one LR image is avaliable. In particular, we focus on *single-frame* SR problems in this paper.

To produce a HR image, the simplest and effective way is to interpolate, e.g., bicubic and nearest interpolations. Recently, more interpolation-based methods have been proposed(see [2–8]). For instance, Youngjoon et al. [8] utilize a generalized curvature source term estimated from the LR image to construct a HR image. In particular, the resulting HR image has a reliable curvature profile which minimizes ringing artifacts. In addition, they propose an iterative application of the curvature interpolation method [9]. The method utilizes the gradient-weighted curvature measured from the LR image, being an interpolator to suppress texture oversmoothing. In [10], Wang et al. present a fast image upsampling method to preserve the sharpness via two-scale sharpness preserving operations. On the one hand, the low-frequency of image is recovered based on a well-constructed displacement field. On the other hand, the local high-frequency structures are reconstructed via a sharpness preserving reconstruction algorithm. However, due to images zooming with the interpolation-based methods to be solved are frequently estimated by information of unknown locations without other priors. The interpolation-based methods usually introduce jagged artifacts or blur effect.

Learning-based methods (see [11–14]) have attracted attention in image processing. Recently, they are also performed impressively in image SR problems. A key issue for the learning-based methods is to learn high frequency correspondences from a database generated by LR and HR image patches pairs. And then we could apply the correspondences to the LR image input to obtain its HR output. Purkait et al. [11] develop fuzzy rules to find different possible HR patches and combine them according to different rule strength to obtain the estimated HR patches. The rule parameters are learned from LR-HR patch pairs and then they use the Takagi-Sugeno (TS) model [15] with the rule parameters expressed as a linear combination of the different input possible HR patches. In addition, Yang et al. [12] apply the theory of sparse coding to SR problems effectively. This method jointly trains two dictionaries for LR and HR image patches, and then they could use the LR dictionary to generate sparse representations of the LR input to obtain the corresponding HR output. And Dong and Loy [13] first learn a mapping between low-resolution and high-resolution images, which is represented by a deep convolutional neural network (CNN). And then they take the LR image as input via the CNN to generate the HR image output. However, these methods typically reply on the similarity between test images and the database. Consequencely, they also involve expensive computation. In recent years, there has been tremendous interest in developing statistics-based methods [14, 16, 17], such as the popular tool Maximum a Posteriori (MAP) and Maximum Likelihood estimator (MLE). As proposed in [14], Peleg and Elad assume that prediction of high resolution patches can be obtained by MMSE estimation and the resulting scheme has the useful interpretation of a feedforward neural network.

Apart from the methods mentioned above, a hybrid method [18], reconstruction methods [19–21], and other methods [22–25] have been used. And these methods are not completely independent with each other. For instance, Peleg and Elad propose a SR method via combining a statistics method and a learning-based method.

In this paper, we study an effective single-frame SR approach, which is an improvement of the so called approximated Heaviside functions method (AHFM) proposed in [7]. It shows that the underlying image, can be viewed as a instensity function, which can be approximately represented by two classes of AHFs. Deng et al. cast the image super-resolution problem as an intensity function estimation problem. Defined on a continuous domain, the underlying intensity image, which belongs to a space with redundant basis, could be approximately represented via two classes of AHFs. Using only one LR image input, we could compute the representation coefficients by the proposed iterative AHF method. Then the high-resolution image is generated by combining the representation coefficients with two classes of AHFs. Here, the two classes of AHFs are corresponding to the high-resolution image.

However, there are only two classes of AHFs, which may not describe/represent the whole information of one image. It may generate some oversharp information for the final HR image. Forced by that, we tend to extend the AHFM algorithm to general form to suppress the resulted image texture oversharp and improve super-resolution images qualities. We consider that an image is normally consisted of different smooth components and non-smooth components. In particular, there are some details, such as edges and corners, which have different sharpness. Based on this, we propose that the smooth components should be represented by multiple classes of AHFs with smooth edges, and the non-smooth components are also represented by multiple classes of AHFs with sharp edges. In particular, due to the sparsity of non-smooth components, we give *l*_1_ regularization model and solve the model via block-wise alternating direction method of multipliers (ADMM) [26]. Furthermore, we design a novel iterative refinement algorithm to pick up more image details. Finally, the proposed method has been numerically proved competitively to some state-of-the-art methods.

The paper is organized as follows. A brief review of the AHFM algorithm [7] is introduced in Section 2. In Section 3, we give the proposed model and its corresponding algorithms. In Section 4, we present the numerical results of different methods. It demonstrates that the proposed algorithms are more efficient. Finally, we conclude the paper in Section 5.

## Some preliminaries

This section gives general remarks on approximated Heaviside functions. In addition, a brief review of the method based on approximated Heaviside functions can be found from [7].

### 2.1 General remarks on Heaviside function

**Fig 1:**
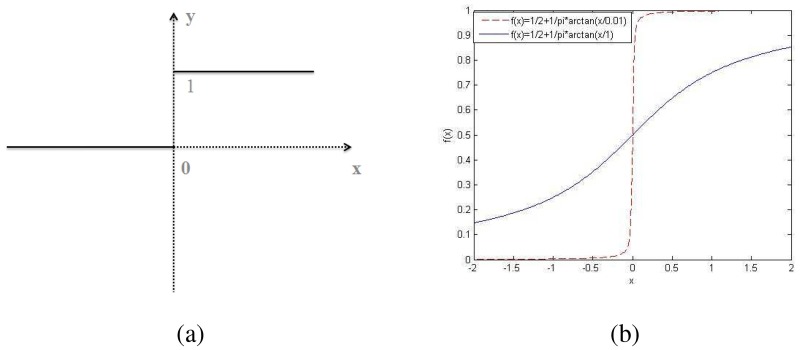
(a) Heaviside function. (b) two approximated Heaviside functions with *ξ* = 1 (blue solid line) and *ξ* = 0.01 (red dash line).

Heaviside function or Heaviside step function, is a discontinuous function whose value is zero for negative argument and one for positive argument. Heaviside function could be defined as following alternative form of the unit step, as a function of a discrete variable *x* (see Fig 1(a)),

$$\begin{array}{c} \phi \left(x\right)=\{\begin{array}{cc} 0 & \;x<0,\;\\ 1 & x\geq 0. \end{array} \end{array}$$ (1)

The definition of *ϕ*(0) = 0 is significant. In the practical applications, some logistic functions to the Heaviside functions are often used for smooth approximations, called approximated Heaviside functions (AHFs), such as

$$\begin{array}{c} \psi \left(x\right)=\frac{1}{1+e^{-2x/\xi }}, \end{array}$$ (2)

or

$$\begin{array}{c} \psi \left(x\right)=\frac{1}{2}+\frac{1}{\pi }\arctan\left(\frac{x}{\xi }\right). \end{array}$$ (3)

As illustrated in Fig 1(b), a smaller *ξ* corresponds to a sharper transition at *x* = 0. In our work, we employ Eq (3) to approximate Heaviside functions.

From [27], Kainen et al. propose that any functions in *L*_*p*_([0, 1]^*d*^), *p* ∈ [1, ∞) could be well approximated by linear combinations of *m* characteristic functions of half-space, and *m* is any positive integer. Let *H*_*d*_ be a set of functions on [0, 1]^*d*^ defined as:

$$\begin{array}{c} H_{d}=\left\{f\;:\;{\left[0,1\right]}^{d}\to R\;:\;f\left(x\right)=\psi \left(v\cdot x+c\right),v\in R^{d},c\in R\right\}, \end{array}$$

where *ψ* is an approximated functions. *H*_*d*_ is the set of characteristic functions of closed half-space of ${ℛ}^{d}$.

**Theorem 1** [27] For any positive integer *d*, define *span*_*m*_*H*_*d*_ as $\left\{\sum_{i=1}^{m}\omega_{i}\psi (v_{i}\cdot x+c)\right\}$, where $\omega_{i}\in ℛ$ and $v_{i}\in {ℛ}^{d}$, and $c_{i}\in ℛ$, then it is known that *U*_*m*∈*n*^+^_*span*_*m*_*H*_*d*_ is dense in (*L*_*p*_([0, 1]^*d*^), ∥ ⋅ ∥_*p*_), *p* ∈ [1, ∞).

**Theorem 2** [27] For any positive integer *m*,*d* and every *p* ∈ [1, ∞), *span*_*m*_*H*_*d*_ is approximately a compact sub-set of (*L*_*p*_([0, 1]^*d*^), ∥ ⋅ ∥_*p*_).

Consequently, we can use *span*_*m*_ *H*_*d*_ for a finite *m* in practical computing.

### 2.2 Single image super-resolution via iterative AHF method (AHFM)

The single image super-resolution via iterative AHF method (AHFM) proposed in [7] for image super-resolution gives a selection of sharp-related terms, which are measured from the LR image input and apply them to fine grids to generate the HR image. They assume the underlying image intensity function *f* is defined on [0, 1]^2^, then *f* ∈ *L*_*p*_([0, 1]^2^) with *p* ∈ [1, ∞). According to the theorems stated in section 2.1, *f* can be approximated by the following equation:

$$\begin{array}{c} f\left(z\right)=\sum_{j=1}^{m}\;\omega_{j}\psi \left(v_{j}\cdot z+c_{j}\right), \end{array}$$ (4)

where $\omega_{j}\in ℛ,v_{j}\in {ℛ}^{2}$, **v** = {(*cosθ*_*t*_, *sinθ*_*t*_)′, *t* = 1, 2, …, *p*} denote *p* different directions, and $c_{j}=\{\frac{1}{q},\frac{2}{q},\frac{3}{q}\ldots ,1\}$ is to denote discrete positions, *m* = *pq*, **z** = (*x*, *y*)′, where *q* is the total number of pixels of the input image. Consequently, the function $\{\psi (v_{j}\cdot z+c_{j}){\}}_{j=1}^{m}$ is called a class of AHF with a specific *ξ*. For an image $L\in {ℛ}^{n_{1}n_{2}}$, we assume it is a discretization of intensity function *f* on [0, 1]^2^, i.e., $L_{i,j}=f(x_{i},y_{j}),x_{i}=\frac{i}{n_{1}},y_{j}=\frac{j}{n_{2}}(i=1,2,\cdots ,n_{1},j=1,2,\cdots ,n_{2})$. Therefore, Eq (4) could be rewritten as matrix-vector form, $L\approx \Psi \omega ,f\in R^{n},\omega \in {ℛ}^{m}$, with *n* = *n*_1_*n*_2_, *m* = *pq*. We compute coefficient *ω* and get the high resolution image with equation $\tilde{\Psi }\omega$, where *s* is an upscaling factor and $\tilde{\Psi }\in {ℛ}^{N\times m}$ with size *N* = *s*^2^*n*_1_*n*_2_.

By this strategy, based on the observation that an image consists of smooth components and non-smooth components. We use two classes of AHFs to depict an image. Different components of an image may be described by different orientations *θ*_*t*_ at the locations *c*_*j*_ with two *ξ*_1_, *ξ*_2_. One is big parameter *ξ*_1_ to represent smooth components (forming Ψ_1_), another one is the smaller *ξ*_2_ to represent non-smooth components (forming Ψ_2_). Thus, the vector-form image *L* can be approximated by the following discrete formula:

$$\begin{array}{c} L\approx \Psi_{1}\beta_{1}+\Psi_{2}\beta_{2}. \end{array}$$ (5)

Since non-smooth components are sparse, *l*_1_ regularization could be given on the coefficient *β*_2_. The optimal model is expressed as:

$$\begin{array}{c} ∥L-\Psi_{1}\beta_{1}-\Psi_{2}\beta_{2}{∥}_{2}^{2}+\;\lambda_{1}∥\beta_{1}{∥}_{2}^{2}+\;\lambda_{2}∥\beta_{2}{∥}_{1}. \end{array}$$ (6)

The Eq (6) is solved via ADMM [28–31] in article [7]. The single image super-resolution via iterative AHF method (AHFM) proposed in [7] is outlined as shown in Algorithm 1.

We close this section with the following remarks.

• In order to pick up more details, such as edges, they design an iterative strategy to conduct an iterative refinement. They consider the difference (*L* − *DH*) as a new low-resolution input of Eq (6) to recompute a residual high-resolution image.

• The AHFM algorithm performs well for natural images. However, the images with smooth backgrounds usually appear ring artifacts along the large scale edges, which mainly come from the added non-smooth components (see step (*b*) in Algorithm 1). Aiming to discard the ring artifacts of non-smooth components *E*, they use bicubic interpolation as the intermediate method to make a mask. Actually, in the step (*b*), only the *E* needs to update by *E*_*new*_, which can be obtained from by the Eq (7), and the ring artifacts could be reduced significantly.

$$\begin{array}{c} E_{new}=\text{Mask}.*E, \end{array}$$ (7)

where

$$\begin{array}{c} \text{Mask}=\{\begin{array}{cc} 0, & \text{if}\;0\leq G_{i,j}\leq t,\\ 1,\; & \text{otherwise}, \end{array} \end{array}$$ (8)

where *G*_*i*, *j*_ is a vector-form of gradient at location (*i*, *j*) of image *B*. The image *B* is generated via the bicubic interpolation. Notation .* stands for dot product. *t* is a threshold value and *t* = 0.05 is in the experiments.

**Algorithm 1** (Single image super-resolution via iterative AHF method (AHFM) [7])

**Input**: one vector-form low-resolution image: $L\in {ℛ}^{n\times 1},\lambda_{1}>0,\lambda_{2}>0$, s: upscaling factor. *τ*: maximum number of iteration.

**Output**: high-resolution image $\hat{H}\in {ℛ}^{N\times 1}$

1. According to Eq (4), construct matrices $\Psi_{1},\Psi_{2}\in {ℛ}^{n\times m}$ on coarse grids, on fine grids construct ${\tilde{\Psi }}_{1},{\tilde{\Psi }}_{2}\in {ℛ}^{N\times m}$, where *N* = *s*^2^*n*.
2. Initialization: *L*^(1)^ = *L*.
  **for k = 1:*τ***
  1. Compute the coefficients:
    $$\begin{array}{c} \left(\beta_{1}^{\left(k\right)},\beta_{2}^{\left(k\right)}\right)=\text{argmin}∥L^{\left(k\right)}-\Psi_{1}\beta_{1}-\Psi_{2}\beta_{2}{∥}_{2}^{2}+\;\lambda_{1}∥\beta_{1}{∥}_{2}^{2}+\;\lambda_{2}∥\beta_{2}{∥}_{1}. \end{array}$$
  2. Update the high-resolution image:
    $$\begin{array}{c} H^{\left(k\right)}=S^{\left(k\right)}+E^{\left(k\right)},\\ \text{where}\;S^{\left(k\right)}={\tilde{\Psi }}_{1}\beta_{1}^{\left(k\right)},E^{\left(k\right)}={\tilde{\Psi }}_{2}\beta_{2}^{\left(k\right)}. \end{array}$$
  3. Downsampling *H*^(*k*)^ to coarse grid: $\tilde{L}=DH^{\left(k\right)}$.
  4. Compute residual: $L^{\left(k+1\right)}=L^{\left(k\right)}-\tilde{L}.$
  **end**
3. Assemble the high-resolution outputs: $S=\sum_{i=1}^{\tau }S^{\left(i\right)},E=\sum_{i=1}^{\tau }E^{\left(i\right)}.$
4. Compute the final high-resolution image:
  $$\begin{array}{c} \hat{H}=S+conv\left(E,p\right), \end{array}$$

where *conv* represents a convolution operator, and *p* is a Gaussian kernel with a small size.

## Modified AHFM and iterative refinement

This section presents a new image super-resolution algorithm, which is an extension of the AHFM algorithm. Note that only two classes of AHFs are not enough for the whole image components. Hence, taking into account the varying sharpness of the whole image, we will consider a modified AHFM with the different sharpness components and further present its algorithm for implementation in next section. Besides, the modified AHFM algorithm contains a new iterative refinement strategy, aiming to pick up more information about non-smooth components.

### 3.1 Modified AHFM with different sharpness components

The AHFM has its respective advantages, which is completely a single image super-resolution method without extra training data. The AHFM algorithm has only used two classes of AHFs to represent the whole image components. The sharper components or the smoother components are pivotal but are not represented well. Motivated by the point and the works proposed in [7], we propose a modified AHFM algorithm to comprise the whole information of one image as well as possible.

First, we assume that a low-resolution image *L* is consisted of smooth components and non-smooth components. We exploit different *ξ* to form different AHFs to describe smooth components and non-smooth components. Hence, the low-resolution image *L* could be approximated by the following discrete formula:

$$\begin{array}{c} L\approx \sum_{i=1}^{l}\;\Psi_{i}\alpha_{i}+\sum_{j=1}^{k}\;\Phi_{j}\beta_{j}, \end{array}$$ (9)

where *l*, *k* represent numbers of smooth components and non-smooth components, respectively. We could obtain $\Psi_{i},\Phi_{j}\in {ℛ}^{n\times m}(i=1,2,\cdots ,l;j=1,2,\cdots ,k)$ according to Eq (4). Ψ_*i*_(*i* = 1, 2, ⋯, *l*) represents smooth components formed by larger *ξ*. Φ_*j*_(*j* = 1, 2, ⋯, *k*) represents non-smooth components formed by smaller *ξ*. And $\alpha_{i},\beta_{j}\in {ℛ}^{m\times 1}(i=1,2,\cdots ,l;j=1,2,\cdots ,k)$ are the corresponding representation coefficients with Ψ_*i*_(*i* = 1, 2, ⋯, *l*) and Φ_*j*_(*j* = 1, 2, ⋯, *k*). After computing the representation coefficients, we apply them into following the equation to obtain the high-resolution image:

$$\begin{array}{c} H\approx \sum_{i=1}^{l}\;{\tilde{\Psi }}_{i}\alpha_{i}+\sum_{j=1}^{k}\;{\tilde{\Phi }}_{j}\beta_{j}, \end{array}$$ (10)

where ${\tilde{\Psi }}_{i},{\tilde{\Phi }}_{j}\in {ℛ}^{N\times m}(i=1,2,\cdots ,l;j=1,2,\cdots ,k;N=s^{2}n)$ are obtained on fine grids. *s* is the upscaling factor.

Since the non-smooth components, such as edges and corner details, are sparse in generic images. Hence, we apply *l*_1_ regularization on *β*_*j*_(*j* = 1, 2, ⋯, *k*) to characterize this feature. Thus, the optimization model can be written as following:

$$\begin{array}{c} \min_{\alpha_{i},\beta_{j}}∥L-\sum_{i=1}^{l}\;\Psi_{i}\alpha_{i}-\sum_{j=1}^{k}\;\Phi_{j}\beta_{j}{∥}_{2}^{2}+\sum_{i=1}^{l}\;\lambda_{i}∥\alpha_{i}{∥}_{2}^{2}+\sum_{j=1}^{k}\;\gamma_{j}∥\beta_{j}{∥}_{1}, \end{array}$$ (11)

where $\lambda_{i},\gamma_{j}\in ℛ(i=1,2,\cdots ,l;j=1,2,\cdots ,k)$ are regularization parameters.

To simplify Eq (11), we set C=(Ψ,Φ), Ψ = (Ψ_1_, Ψ_2_, ⋯, Ψ_*l*_), Φ = (Φ_1_, Φ_2_, ⋯, Φ_*k*_), *u* = (*α*_1_, *α*_2_, ⋯, *α*_*l*_, *β*_1_, *β*_2_, ⋯, *β*_*k*_)^*T*^,

$$\begin{array}{c} M_{i}=\left(\overset{l}{\overset{︷}{0,\cdots ,0,\underset{ith}{\underset{︸}{I}},\cdots ,0}},\underset{k}{\underset{︸}{0,\cdots ,0}}\right),B_{j}=\left(\underset{l}{\underset{︸}{0,0,\cdots ,0}},\overset{k}{\overset{︷}{0,\cdots ,\underset{jth}{\underset{︸}{I}},\cdots ,0}}\right). \end{array}$$

Thus, Eq (11) could be rewritten as following:

$$\begin{array}{c} \min_{u}∥L-Cu{∥}_{2}^{2}+\sum_{i=1}^{l}\;\lambda_{i}∥M_{i}u{∥}_{2}^{2}+\sum_{j=1}^{k}\;\gamma_{j}∥B_{j}u{∥}_{1}. \end{array}$$ (12)

As *l*_1_ term is not differentiable, we make a set of variable substitutions for *B*_*j*_*u*(*j* = 1, 2, ⋯, *k*) and then rewrite Eq (12) as:

$$\begin{array}{c} \min_{u}∥L-Cu{∥}_{2}^{2}+\sum_{i=1}^{l}\;\lambda_{i}∥M_{i}u{∥}_{2}^{2}+\sum_{j=1}^{k}\;\gamma_{j}∥p_{j}{∥}_{1},\\ s.t.,p_{j}=B_{j}u\left(j=1,2,\cdots ,k\right), \end{array}$$ (13)

where *p*_*j*_(*j* = 1, 2, ⋯, *k*) are the substitution variable. In particular, the Eq (13) is separable, w.r.t (*u*, *p*_*j*_)(*j* = 1, 2, ⋯, *k*). Many methods are used to solve the *l*_1_ problem (see [28–33]). The work in [7] solve the *l*_1_ problem via alternating direction method of multipliers(ADMM) [31]. Here, we solve Eq (13) via block-wise ADMM [26].

The augmented Lagrangian of Eq (13) is

$$\begin{array}{c} L\left(u,p_{j},b_{j}\right)=∥L-Cu{∥}_{2}^{2}+\sum_{i=1}^{l}\;\lambda_{i}∥M_{i}u{∥}_{2}^{2}+\sum_{j=1}^{k}\;\gamma_{j}∥p_{j}{∥}_{1}+\sum_{j=1}^{k}\;\frac{\tau_{j}}{2}∥p_{j}-B_{j}u+b_{j}{∥}_{2}^{2}, \end{array}$$ (14)

where *b*_*j*_(*j* = 1, 2, ⋯, *k*) are Lagrangian multipliers with proper size. The problem of minimizing $ℒ(u,p_{j},b_{j})$ could be solved by iteratively and alternatively via the following subproblems:

$$\begin{array}{c} u\text{-}subproblem\;:\;\min_{u}∥L-Cu{∥}_{2}^{2}+\sum_{i=1}^{l}\;\lambda_{i}∥M_{i}u{∥}_{2}^{2}+\sum_{j=1}^{k}\;\frac{\tau_{j}}{2}∥p_{j}-B_{j}u+b_{j}{∥}_{2}^{2}, \end{array}$$ (15)

$$\begin{array}{c} p_{j}\text{-}subproblem\;:\;\text{min}\gamma_{j}∥p_{j}{∥}_{1}+\frac{\tau_{j}}{2}∥p_{j}-B_{j}u+b_{j}{∥}_{2}^{2}\left(j=1,2,\cdots ,k\right). \end{array}$$ (16)

Hence, the solution to the problem (11) is solved by block-wise ADMM, which is shown in Algorithm 2:

**Algorithm** **2**

**Input**: Given the low-resolution image $L\in {ℛ}^{n\times 1}$, $\Psi_{i},\Phi_{j}\in {ℛ}^{n\times m}$, λ_*i*_(*i* = 1, 2, ⋯, *l*), *γ*_*j*_ > 0, *τ*_*j*_ > 0(*j* = 1, 2, ⋯, *k*)

**Output**: coefficient *u*

1. **Initialization**: $u^{\left(1\right)}\leftarrow 0,p_{j}^{\left(1\right)}\leftarrow 0,b_{j}^{\left(1\right)}\leftarrow 0(j=1,2,\cdots ,k)$
2. ***while not converged do***
3.  *t* ← *t* + 1
4.  *u*^(*t*)^ ← solve subproblem (15) for $p_{j}=p_{j}^{\left(t-1\right)},b_{j}=b_{j}^{\left(t-1\right)}$
5.  $p_{j}^{\left(t\right)}$ ← solve subproblem (16) for $u=u^{\left(t\right)},b_{j}=b_{j}^{\left(t-1\right)}$
6.  $b_{j}^{\left(t\right)}\leftarrow b_{j}^{\left(t\right)}+(p_{j}^{\left(t\right)}-B_{j}u^{\left(t\right)})$.
7. ***end while***

The *u-subproblem* is a smooth quadratic problem, we can solve it by least squares method:

$$\begin{array}{c} u=K^{-1}q, \end{array}$$ (17)

where $u\in {ℛ}^{(k+l)m\times 1}$,

$$\begin{array}{c} K=C^{T}C+\sum_{i=1}^{l}\;\lambda_{i}M_{i}^{T}M_{i}+\sum_{j=1}^{k}\;\frac{\tau_{j}}{2}B_{j}^{T}B_{j}\in R^{(k+l)m\times (k+l)m},\\ q=C^{T}L+\sum_{j=1}^{k}\;\frac{\tau_{j}}{2}\left(B_{j}^{T}p_{j}+B_{j}^{T}b_{j}\right)\in R^{(k+l)m\times 1}. \end{array}$$

The *p*_*j*_-*subproblem*(*j* = 1, 2, ⋯, *k*) has a closed form solution for each (*p*_*j*_)_*i*_ (see [33]),

$$\begin{array}{c} {\left(p_{j}\right)}_{i}=shrink\left({\left(B_{j}u\right)}_{i}-{\left(b_{j}\right)}_{i},\frac{\gamma_{j}}{\tau_{j}}\right), \end{array}$$ (18)

where *shrink*(*a*, *b*) = sign(*a*) max(|*a*| − *b*, 0) and 0.(0/0) = 0 is assumed.

By Algorithm 2, we have computed the representation coefficients *α*_*i*_, *β*_*j*_(*i* = 1, 2, ⋯, *l*; *j* = 1, 2, ⋯, *k*). We can get the high resolution image $\hat{H}$ via Eq (10).

### 3.2 Modified AHFM with iterative refinement

The Eq (9) takes different smooth components and non-smooth components into consideration. A natural remedy for extracting more details is to find the structure of the difference between LR image and last updated resulted image. We find that residual $R=L-D\hat{H}$, where $\hat{H}$ is the last updated HR image and *D* is the downsampled operator. Fortunately, we find the residual is mostly non-smooth components. To pick up more edge details to make image less blurry, we design a new iterative refinement model based on the special structure. In the model, we use two smaller $\xi_{3}^{*},\xi_{4}^{*}$ (forming $\Phi_{3}^{*},\Phi_{4}^{*}$) to depict the residual image. Since the non-smooth components are also sparse in the residual. We apply *l*_1_ regularization to the corresponding coefficients. The iterative refinement model could be written as following:

$$\begin{array}{c} \min_{\beta_{3}^{*},\beta_{4}^{*}}∥R-\Phi_{3}^{*}\beta_{3}^{*}-\Phi_{4}^{*}\beta_{4}^{*}{∥}_{2}^{2}+\mu_{1}∥\beta_{3}^{*}{∥}_{1}+\mu_{2}∥\beta_{4}^{*}{∥}_{1}. \end{array}$$ (19)

$\beta_{3}^{*},\beta_{4}^{*}$ are the coefficients of the non-smooth components. *μ*_1_, *μ*_2_ are regularization parameters. Since the Eq (19) is the form of *l*_1_ norm, we solve it by ADMM scheme. We make two variable substitutions for $\beta_{3}^{*},\beta_{4}^{*}$, and rewrite Eq (19) as:

$$\begin{array}{c} \min_{\beta_{3}^{*},\beta_{4}^{*}}∥R-\Phi_{3}^{*}\beta_{3}^{*}-\Phi_{4}^{*}\beta_{4}^{*}{∥}_{2}^{2}+\mu_{1}∥u_{1}{∥}_{1}+\mu_{2}∥u_{2}{∥}_{1},\\ s.t.,u_{1}=\beta_{3}^{*},u_{2}=\beta_{4}^{*}. \end{array}$$ (20)

We rewrite Eq (20) as:

$$\begin{array}{c} \min_{\beta^{*}}∥R-D\beta^{*}{∥}_{2}^{2}+\mu_{1}∥u_{1}{∥}_{1}+\mu_{2}∥u_{2}{∥}_{1},\\ s.t.,u_{1}=A\beta^{*},u_{2}=B\beta^{*}, \end{array}$$ (21)

where $D=(\Phi_{3}^{*},\Phi_{4}^{*}),\beta^{*}=(\beta_{3}^{*},\beta_{4}^{*}{)}^{T}$, *A* = (*I*, **0**), and *B* = (**0**, *I*). And **0** is zero matrix. *I* is identity matrix. Since the optimization Eq (21) is separable, w.r.t (β*, *u*_1_, *u*_2_). The augmented Lagrangian of Eq (21) is

$$\begin{array}{c} \begin{array}{cc} L_{{\tilde{\tau }}_{1},{\tilde{\tau }}_{2}}\left(\beta^{*},u_{1},u_{2},b_{1}^{*},b_{2}^{*}\right) & =∥R-D\beta^{*}{∥}_{2}^{2}+\mu_{1}∥u_{1}{∥}_{1}+\mu_{2}∥u_{2}{∥}_{1}\\ & +\frac{{\tilde{\tau }}_{1}}{2}∥u_{1}-A\beta^{*}+b_{1}^{*}{∥}_{2}^{2}+\frac{{\tilde{\tau }}_{2}}{2}∥u_{2}-B\beta^{*}+b_{2}^{*}{∥}_{2}^{2}, \end{array} \end{array}$$ (22)

where $b_{1}^{*},b_{2}^{*}$ are the proper size Lagrangian multipliers. By ADMM, the minimizing of Eq (22) is solved by following three subproblems:

$$\begin{array}{c} \beta^{*}\text{-}subproblem\;:\;\min_{\beta^{*}}∥R-D\beta^{*}{∥}_{2}^{2}+\frac{{\tilde{\tau }}_{1}}{2}∥u_{1}-A\beta^{*}+b_{1}^{*}{∥}_{2}^{2}+\frac{{\tilde{\tau }}_{2}}{2}∥u_{2}-B\beta^{*}+b_{2}^{*}{∥}_{2}^{2}, \end{array}$$ (23)

$$\begin{array}{c} u_{1}\text{-}subproblem\;:\;\min_{u_{1}}\mu_{1}∥u_{1}{∥}_{1}+\frac{{\tilde{\tau }}_{1}}{2}∥u_{1}-A\beta^{*}+b_{1}^{*}{∥}_{2}^{2}, \end{array}$$ (24)

$$\begin{array}{c} u_{2}\text{-}subproblem\;:\;\min_{u_{2}}\mu_{2}∥u_{2}{∥}_{1}+\frac{{\tilde{\tau }}_{2}}{2}∥u_{2}-B\beta^{*}+b_{2}^{*}{∥}_{2}^{2}. \end{array}$$ (25)

We could use least squares method to solve β*-*subproblem*:

$$\begin{array}{c} \beta^{*}={\left(K_{1}\right)}^{-1}q_{1}, \end{array}$$ (26)

where $\beta^{*}\in {ℛ}^{2m\times 1}$, $K_{1}=2D^{T}D+{\tilde{\tau }}_{1}A^{T}A+{\tilde{\tau }}_{2}B^{T}B\in {ℛ}^{2m\times 2m}$ and $q_{1}=2D^{T}R+{\tilde{\tau }}_{1}A^{T}(u_{1}+b_{1}^{*})+{\tilde{\tau }}_{2}B^{T}(u_{2}+b_{2}^{*})\in {ℛ}^{2m\times 1}.$ The subproblem *u*_1_ and *u*_2_ have a closed form solution as mentioned in Algorithm 2. Thus, the solutions to the subproblems *u*_1_ and *u*_2_ are shown as following:

$$\begin{array}{c} {\left(u_{1}\right)}_{j}=shrink\left({\left(A\beta^{*}\right)}_{j}-{\left(b_{1}^{*}\right)}_{j},\frac{\mu_{1}}{{\tilde{\tau }}_{1}}\right), \end{array}$$ (27)

$$\begin{array}{c} {\left(u_{2}\right)}_{j}=shrink\left({\left(B\beta^{*}\right)}_{j}-{\left(b_{2}^{*}\right)}_{j},\frac{\mu_{2}}{{\tilde{\tau }}_{2}}\right). \end{array}$$ (28)

The main algorithm for iterative refinement is shown in Algorithm 3. We will illustrate the necessary of the Algorithm 3 in section 4.

**Algorithm 3** (Iterative refinement)

**Input**: residual image *R*, *μ*_1_, *μ*_2_, ${\tilde{\tau }}_{1}$, ${\tilde{\tau }}_{2}$,$\Psi_{3}^{*},\Psi_{4}^{*}\in {ℛ}^{n\times m}$

**Output**: coefficient *β*

1. **Initialization**: $\beta^{\left(1\right)}\leftarrow 0,\mu_{1}^{\left(1\right)}\leftarrow 0,\mu_{2}^{\left(1\right)}\leftarrow 0,b_{1}^{*(1)}\leftarrow 0,b_{2}^{*(1)}\leftarrow 0$
2. ***while not converged do***
3.  *t* ← *t* + 1
4.  β*^(*t*)^ ← solve subproblem (23) for $u_{1}=u_{1}^{\left(t-1\right)},u_{2}=u_{2}^{\left(t-1\right)}$,
  $$\begin{array}{c} b_{1}^{*}=b_{1}^{*(t-1)},b_{2}^{*}=b_{2}^{*(t-1)}, \end{array}$$
5.  $u_{1}^{\left(t\right)}$ ← solve subproblem (24) for $\beta^{*}=\beta^{*(t)},b_{1}^{*}=b_{1}^{*(t-1)},$
6.  $u_{2}^{\left(t\right)}$ ← solve subproblem (25) for $\beta^{*}=\beta^{*(t)},b_{2}^{*}=b_{2}^{*(t-1)},$
7.  $b_{1}^{*(t)}\leftarrow b_{1}^{*(t)}=b_{1}^{*(t-1)}+(u_{1}^{\left(t\right)}-A\beta^{*(t)}),$
8.  $b_{2}^{*(t)}\leftarrow b_{2}^{*(t)}=b_{2}^{*(t-1)}+(u_{2}^{\left(t\right)}-A\beta^{*(t)}).$
9. ***end while***

### 3.3 Single image super-resolution based on approximated Heaviside functions and iterative refinement

**Fig 2:**
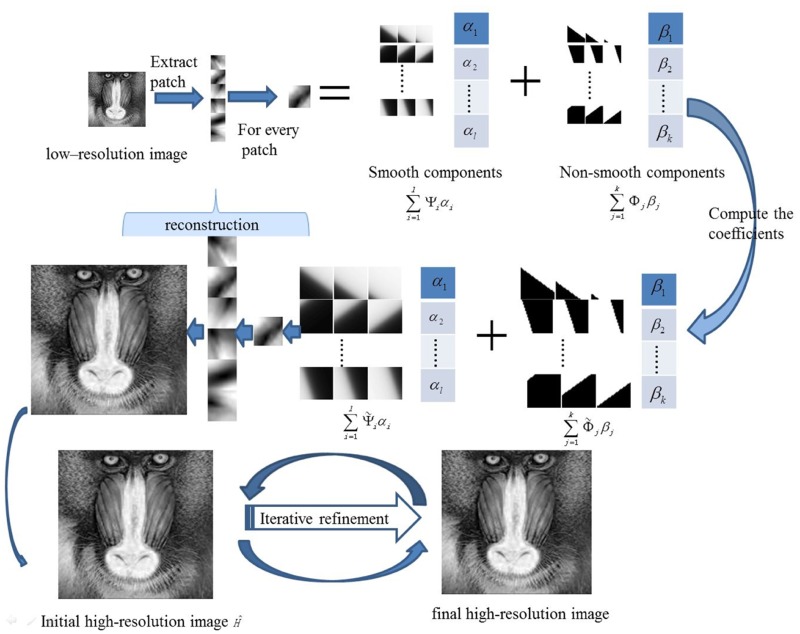
The flow chart of the proposed work.

Take different behaviours of Eqs (9) and (19) into consideration, we get the modified single super-resolution based on approximated Heaviside functions and iterative refinement, which is shown in Algorithm 4. The details of the proposed method could be found from the flow chart shown in the Fig 2.

**Algorithm** **4**

**Input**: Given the low-resolution image L (a vector form), λ_*i*_, *γ*_*j*_ > 0(*i* = 1, 2, ⋯, *l*;*j* = 1, 2, ⋯, *k*), *μ*_1_ > 0, *μ*_2_ > 0, ${\tilde{\tau }}_{1}>0$, ${\tilde{\tau }}_{2}>0$. *s*: the upscaling factor. *t*: maximum iterations of the iterative refinement.

**Output**: high-resolution *H*

1. According to Eq (4), construct matrices $\Psi_{i},\Phi_{j}\in {ℛ}^{n\times m}$, $\Phi_{3}^{*},\Phi_{4}^{*}\in {ℛ}^{n\times m}$ on coarse grids, construct ${\tilde{\Psi }}_{i},{\tilde{\Phi }}_{j}(i=1,2,\cdots ,l;j=1,2,\cdots ,k){\tilde{\Phi }}_{3}^{*},{\tilde{\Phi }}_{4}^{*}\in {ℛ}^{N\times m}$ on corresponding fine grids where *N* = *s*^2^*n*.
2. Compute the coefficients (*α*_*i*_, *β*_*j*_) according to Algorithm 2:
  $$\begin{array}{c} \left(\alpha_{i},\beta_{j}\right)=∥L-\sum_{i=1}^{l}\;\Psi_{i}\alpha_{i}-\sum_{j=1}^{k}\;\Phi_{j}\beta_{j}{∥}_{2}^{2}+\sum_{i=1}^{l}\;\lambda_{i}∥\alpha_{i}{∥}_{2}^{2}+\sum_{j=1}^{k}\;\gamma_{j}∥\beta_{j}{∥}_{1}. \end{array}$$
3. Get high-resolution image $\hat{H}$: $\hat{H}=\sum_{i=1}^{l}S_{i}+\sum_{j=1}^{k}E_{j},S_{i}={\tilde{\Psi }}_{i}\alpha_{i},E_{j}={\tilde{\Phi }}_{j}\beta_{j}.$
4. **Initialization**: $H^{\left(1\right)}=\hat{H}$.
  **for *k* = 1, 2, ⋯, *t***
  1. Compute the residual image *R*^(*k*)^: *R*^(*k*)^ = *L* − *DH*^(*k*)^.
  2. Compute the coefficients according to Algorithm 3:
    $$\begin{array}{c} \left(\beta_{3}^{*(k)},\beta_{4}^{*(k)}\right)=\text{argmin}∥R^{\left(k\right)}-\Phi_{3}^{*}\beta_{3}^{*}-\Phi_{4}^{*}\beta_{4}^{*}{∥}_{2}^{2}+\mu_{1}∥\beta_{3}^{*}{∥}_{1}+\mu_{2}∥\beta_{4}^{*}{∥}_{1}. \end{array}$$
  3. Update the high-resolution image:
    $$\begin{array}{c} H^{\left(k+1\right)}=H^{\left(k\right)}+E_{1}^{\left(k\right)}+E_{2}^{\left(k\right)},\\ \text{where}\;E_{1}^{\left(k\right)}={\tilde{\Phi }}_{3}^{*}\beta_{3}^{*(k)},E_{2}^{\left(k\right)}={\tilde{\Phi }}_{4}^{*}\beta_{4}^{*(k)}. \end{array}$$
  **end**
5. Get non-smooth components: $E_{1}=\sum_{i=1}^{t}E_{1}^{\left(i\right)},E_{2}=\sum_{i=1}^{t}E_{2}^{\left(i\right)}.$
6. Compute the final high-resolution image: $H=\hat{H}+conv(E_{1},p)+conv(E_{2},p),$ where *conv* represents a convolution operator, *p* is a Gaussian kernel with a small size.

We use a Gaussian kernel *p* with a small size to make a convolution to avoid the oversharp information on the non-edge parts. And *D* is the bicubic downsampling operator.

However, the computation of the Algorithm 4 is very expensive, due to the large scale and non-sparse matrix C in Eq (12). For example, if a LR image is 512 × 512 and we choose 10 different directions, the size of matrix C will be equal to (512^2^ × 10 × 512^2^). It is obviously large. Thus, it is observed that if we increase the number of smooth components and non-smooth components. The quality of an image usually gets improved at the cost of increased computation time and memory requirement. However, for large number of smooth components and non-smooth components, it is difficult to do SR on a limited hardware. We have experimentally seen that the use of more than *l* = 1, *k* = 2 in Algorithm 4 does not improve quality of the images significantly. In addition, *l* = 1, *k* = 2 work well on a regular desktop. Hence, we choose *l* = 1, *k* = 2 in our work. The optimization model can be simplified as:

$$\begin{array}{c} \min_{\alpha_{1},\beta_{1},\beta_{2}}∥L-\Psi_{1}\alpha_{1}-\Phi_{1}\beta_{1}-\Phi_{2}\beta_{2}{∥}_{2}^{2}+\lambda_{1}∥\alpha_{1}{∥}_{2}^{2}+\lambda_{2}∥\beta_{1}{∥}_{1}+\lambda_{3}∥\beta_{2}{∥}_{1}. \end{array}$$ (29)

To simplify Eq (29), we set $\tilde{C}=(\Psi_{1},\Phi_{1},\Phi_{2})$, $\tilde{u}=(\alpha_{1},\beta_{1},\beta_{2})$, *M* = (**I**, **0**, **0**), $B_{1}^{{}^{\prime }}=(0,I,0)$, $B_{2}^{{}^{\prime }}=(0,0,I)$, where **0** is zero matrix, **I** is identity matrix. The Eq (29) can be rewritten as following.

$$\begin{array}{c} \min_{\tilde{u}}∥L-\tilde{C}\tilde{u}{∥}_{2}^{2}+\lambda_{1}∥M\tilde{u}{∥}_{2}^{2}+\gamma_{1}∥B_{1}^{{}^{\prime }}\tilde{u}{∥}_{1}+\gamma_{2}∥B_{2}^{{}^{\prime }}\tilde{u}{∥}_{1}. \end{array}$$ (30)

Since *l*_1_ term is not differentiable, we make two variable substitutions *p*_1_, *p*_2_ for $B_{1}^{{}^{\prime }}\tilde{u},B_{2}^{{}^{\prime }}\tilde{u}$ to rewrite the Eq (30) as:

$$\begin{array}{c} \min_{\tilde{u}}∥L-\tilde{C}\tilde{u}{∥}_{2}^{2}+\lambda_{1}∥M\tilde{u}{∥}_{2}^{2}+\gamma_{1}∥p_{1}{∥}_{1}+\gamma_{2}∥p_{2}{∥}_{1},\\ s.t.,p_{1}=B_{1}^{{}^{\prime }}\tilde{u},p_{2}=B_{2}^{{}^{\prime }}\tilde{u}. \end{array}$$ (31)

The Eq (31) is separable, w.r.t $\left(\tilde{u},p_{1},p_{2}\right)$. Here, we solve this problem by block-wise ADMM.

The augmented Lagrangian of Eq (31) is

$$\begin{array}{c} \begin{array}{cc} L(\tilde{u},p_{1},p_{2},b_{1},b_{2})= & ∥L-\tilde{C}\tilde{u}{∥}_{2}^{2}+\lambda_{1}∥M\tilde{u}{∥}_{2}^{2}+\gamma_{1}∥p_{1}{∥}_{1}+\gamma_{2}∥p_{2}{∥}_{1}\\ & +\frac{\tau_{1}}{2}∥p_{1}-B_{1}^{{}^{\prime }}\tilde{u}+b_{1}{∥}_{2}^{2}+\frac{\tau_{2}}{2}∥p_{2}-B_{2}^{{}^{\prime }}\tilde{u}+b_{2}{∥}_{2}^{2}, \end{array} \end{array}$$ (32)

where *b*_1_, *b*_2_ are Lagrangian multipliers with proper size. The problem of minimizing $ℒ(\tilde{u},p_{1},p_{2},b_{1},b_{2})$ could be solved by iteratively and alternatively by following subproblems:

$$\begin{array}{c} \begin{array}{cc} \tilde{u}\text{-}subproblem\;:\;\text{min}∥L-\tilde{C}\tilde{u}{∥}_{2}^{2}+\lambda_{1}∥M\tilde{u}{∥}_{2}^{2} & +\frac{\tau_{1}}{2}∥p_{1}-B_{1}^{{}^{\prime }}\tilde{u}+b_{1}{∥}_{2}^{2}\\ & +\frac{\tau_{2}}{2}∥p_{2}-B_{2}^{{}^{\prime }}\tilde{u}+b_{2}{∥}_{2}^{2}, \end{array} \end{array}$$ (33)

$$\begin{array}{c} p_{1}\text{-}subproblem\;:\;\text{min}\gamma_{1}∥p_{1}{∥}_{1}+\frac{\tau_{1}}{2}∥p_{1}-B_{1}^{{}^{\prime }}\tilde{u}+b_{1}{∥}_{2}^{2}, \end{array}$$ (34)

$$\begin{array}{c} p_{2}\text{-}subproblem\;:\;\text{min}\gamma_{2}∥p_{2}{∥}_{1}+\frac{\tau_{2}}{2}∥p_{2}-B_{2}^{{}^{\prime }}\tilde{u}+b_{2}{∥}_{2}^{2}. \end{array}$$ (35)

The $\tilde{u}\text{-}subproblem$ is a smooth quadratic problem. We solve it by least squares method as following:

$$\begin{array}{c} \tilde{u}=K_{1}^{-1}q_{1}, \end{array}$$ (36)

where

$$\begin{array}{c} K_{1}={\tilde{C}}^{T}\tilde{C}+\lambda_{1}M^{T}M+\frac{\tau_{1}}{2}{\left(B_{1}^{{}^{\prime }}\right)}^{T}B_{1}+\frac{\tau_{2}}{2}{\left(B_{2}^{{}^{\prime }}\right)}^{T}B_{2}, \end{array}$$ (37)

$$\begin{array}{c} q_{1}={\tilde{C}}^{T}L+\sum_{j=1}^{2}\;\frac{\tau_{j}}{2}{\left(B_{j}^{{}^{\prime }}\right)}^{T}\left(p_{j}+b_{j}\right). \end{array}$$ (38)

The *p*_*j*_-*subproblem*(*j* = 1, 2) has a closed form solution Eqs (39) and (40) for each (*p*_*j*_)_*i*_(*j* = 1, 2):

$$\begin{array}{c} {\left(p_{1}\right)}_{i}=shrink\left({\left(B_{1}^{{}^{\prime }}\tilde{u}\right)}_{i}-{\left(b_{1}\right)}_{i},\frac{\gamma_{1}}{\tau_{1}}\right), \end{array}$$ (39)

$$\begin{array}{c} {\left(p_{2}\right)}_{i}=shrink\left({\left(B_{2}^{{}^{\prime }}\tilde{u}\right)}_{i}-{\left(b_{2}\right)}_{i},\frac{\gamma_{2}}{\tau_{2}}\right), \end{array}$$ (40)

where *shrink*(*a*, *b*) = sign(*a*) max(|*a*| − *b*, 0).

We summarize *l* = 1, *k* = 2 in Algorithm 4 as modified single image based on approximated Heaviside functions and iterative refinement, which we call this algorithm as MAHFM, shown in Algorithm 5.

**Algorithm 5** (MAHFM algorithm)

**Input**: Given the low-resolution image L (vector form), λ_1_, *γ*_1_, *γ*_2_ > 0, *μ*_1_ > 0, *μ*_2_ > 0, ${\tilde{\tau }}_{1}>0$, ${\tilde{\tau }}_{2}>0$, *s*: the upscaling factor. *t*: maximum iterations of the iterative refinement.

**Output**: high-resolution *H*

1. According to Eq (4), construct matrices $\Psi_{1},\Phi_{1},\Phi_{2},\Phi_{3}^{*},\Phi_{4}^{*}\in {ℛ}^{n\times m}$ on coarse grids, construct ${\tilde{\Psi }}_{1},{\tilde{\Phi }}_{1},{\tilde{\Phi }}_{2},{\tilde{\Phi }}_{3}^{*},{\tilde{\Phi }}_{4}^{*}\in {ℛ}^{N\times m}$ on corresponding fine grids where *N* = *s*^2^*n*.
2. Compute the coefficients according to Algorithm 2:
  $$\begin{array}{c} \left(\alpha_{1},\beta_{1},\beta_{2}\right)=\text{argmin}∥L-\Psi_{1}\alpha_{1}-\Phi_{1}\beta_{1}-\Phi_{2}\beta_{2}{∥}_{2}^{2}+\lambda_{1}∥\alpha_{1}{∥}_{2}^{2}+\gamma_{1}∥\beta_{1}{∥}_{1}+\gamma_{2}∥\beta_{2}{∥}_{1}. \end{array}$$
3. Get high-resolution image $\hat{H}$: $\hat{H}=S_{1}+\sum_{j=1}^{2}E_{j},S_{1}={\tilde{\Psi }}_{1}\alpha_{1},E_{j}={\tilde{\Phi }}_{j}\beta_{j}(j=1,2).$
4. **Initialization**: $H^{\left(1\right)}=\hat{H}$.
  **for *k* = 1, 2, ⋯, *t***
  1. Compute the residual image *R*^(*k*)^: *R*^(*k*)^ = *L* − *DH*^(*k*)^.
  2. Compute the coefficients according to Algorithm 3:
    $$\begin{array}{c} \left(\beta_{3}^{*(k)},\beta_{4}^{*(k)}\right)=\text{argmin}∥R^{\left(k\right)}-\Phi_{3}^{*}\beta_{3}^{*}-\Phi_{4}^{*}\beta_{4}^{*}{∥}_{2}^{2}+\mu_{1}∥\beta_{3}^{*}{∥}_{1}+\mu_{2}∥\beta_{4}^{*}{∥}_{1}. \end{array}$$
  3. Update the high-resolution image:
    $$\begin{array}{c} H^{\left(k+1\right)}=H^{\left(k\right)}+E_{1}^{\left(k\right)}+E_{2}^{\left(k\right)},\\ \text{where}\;E_{1}^{\left(k\right)}={\tilde{\Phi }}_{3}^{*}\beta_{3}^{*(k)},E_{2}^{\left(k\right)}={\tilde{\Phi }}_{4}^{*}\beta_{4}^{*(k)}. \end{array}$$
  **end**
5. Get non-smooth components: $E_{1}=\sum_{i=1}^{t}E_{1}^{\left(i\right)},E_{2}=\sum_{i=1}^{t}E_{2}^{\left(i\right)}.$
6. Compute the final high-resolution image: $H=\hat{H}+conv(E_{1},p)+conv(E_{2},p),$ where *conv* represents a convolution operator, *p* is a Gaussian kernel with a small size.

## Experiments

### 4.1 Numerical results

We have implemented our algorithm to some benchmark images whose high-resolution versions are available. For a quantitative comparison, we downscale actual HR images to their LR versions via bicubic interpolation and then generate HR images by MAHFM algorithm and other methods. For gray images, we perform the proposed algorithm to them directly. While working for color images, the input image is first converted from RGB to YCbCr. We only perform the MAHFM algorithm on the illuminance channel because the human are more sensitive to the brightness information. The other two channels contain chromaticity information and they could be upsampled by bicubic interpolation. Finally, we convert three channels back to RGB to get the estimated color HR image.

We make the quantitative comparisons between the recovered HR images and the actual HR images. And we use root mean square error (RMSE) and peak signal to noise ratio (PSNR) on the illuminance channel to evaluate numerical performance.

$$\begin{array}{c} \text{RMSE}=\sqrt{\frac{1}{N}\;\sum_{i=1}^{N}{\left(h_{i}-{\hat{h}}_{i}\right)}^{2}}, \end{array}$$ (41)

$$\begin{array}{c} \text{PSNR}=10\cdot {\text{log}}_{10}(\frac{{\left(2^{n}-1\right)}^{2}}{\frac{1}{IJ}\sum_{i,j}{\left(h_{ij}-{\hat{h}}_{ij}\right)}^{2}}), \end{array}$$ (42)

where *h*, $\hat{h}$ are the vector-form of ground-truth image and the resulted high-resolution image, respectively. *N* represents testing times for one image. *IJ* is the original image consisting of (*I* × *J*) pixels. *n* is the number of bits per sample. The smaller RMSE is, the better performances of the method usually are. But PSNR has the opposite property. As mentioned in [7], if we apply MAHFM algorithm to one image, it would involve expensive computation. It follows that we could utilize image patches to reduce computation and storage significantly. In our work, we set patch size to be 6 × 6 and overlap to be 3.

First, we compare the MAHFM algorithm with AHFM algorithm [7], and the numerical results are shown in Table 1. The performance of MAHFM algorithm is compared with that of bicubic interpolation, a kernel regression method (denoted as “07’TIP” [34]), a fast upsampling method (denoted as “08’TOG” [22]), one state-of-the art learning-based method (denoted as “10’TIP” [12]), a fast image upsampling method via the displacement field (denoted as “14’TIP” [10]) and AHFM [7]. The numerical results by these methods are displayed in Table 2. Furthermore, we have also carried out our experiments for the upscaling factor *s* = 3. The quantitative measurements for them are shown in Table 3. As can be seen, these results reveal that the MAHFM algorithm is effective.

**Table 1:** Comparison with AHFM algorithm and MAHFM algorithm(factor = 2).

Algorithms	PSNR/RMSE	*woodpecker*	*workman*	*flowers*	*butterfly*	*children*	*blackbutterfly*
AHFM	RMSE	3.3077	5.5733	10.3057	11.7942	3.7469	6.4146
	PSNR	38.4155	33.2085	27.8692	26.6974	036.6573	31.9874
MAHFM	RMSE	**3.0950**	**5.4714**	**6.7661**	**11.2689**	**3.5336**	**6.2337**
	PSNR	**38.6273**	**33.3689**	**31.5241**	**27.0932**	**37.1665**	**32.2359**

**Table 2:** Quantitative results by different super-resolution algorithms (factor = 2).

Images	PSNR/RMSE	bicubic	07’TIP [34]	08’TOG [22]	10’TIP [12]	AHFM [7]	MAHFM
*leaves*	RMSE	15.6427	16.5913	16.1366	15.1104	14.8658	**14.8190**
	PSNR	24.2446	23.7332	23.9746	24.5433	24.6871	**24.7145**
*father*	RMSE	6.1870	8.5823	6.8589	5.6152	5.6024	**5.5610**
	PSNR	32.3012	29.4587	31.4057	33.1435	33.1633	**33.2354**
*comic*	RMSE	12.6995	16.4580	13.9571	11.1757	11.5335	**11.1000**
	PSNR	26.0551	23.8033	25.2349	27.1653	26.8916	**27.2243**
*babyface*	RMSE	4.6689	5.9649	5.1108	4.4681	4.4029	**4.3713**
	PSNR	34.7466	32.6188	33.9610	35.1284	35.2560	**35.3185**
*lions*	RMSE	7.3928	10.4452	8.2584	6.7658	6.4465	**6.2582**
	PSNR	30.7546	27.7524	29.7929	31.5244	31.9444	**32.2018**
*tree*	RMSE	13.2687	15.8486	13.8727	12.7048	12.4237	**12.3883**
	PSNR	25.6742	24.1310	25.2876	26.0515	26.2458	**26.2705**
*peppers*	RMSE	5.6703	7.1575	5.9815	5.1910	4.9485	**4.6897**
	PSNR	33.0587	31.0356	32.5945	33.8258	34.2413	**34.7080**

**Table 3:** Quantitative results by different super-resolution algorithms (factor = 3).

Images	PSNR/RMSE	07’TIP [34]	08’TOG [22]	10’TIP [12]	14’TIP [10]	AHFM [7]	MAHFM
*barbara*	RMSE	15.7546	11.9587	12.2959	22.1094	12.0127	**11.8880**
	PSNR	24.1826	26.5771	26.3356	21.2393	26.1975	**26.6286**
*view*	RMSE	14.4619	**10.9190**	11.2941	21.9687	11.3169	11.2920
	PSNR	24.9263	**27.3672**	27.0731	21.2947	27.0562	27.0754
*baboon*	RMSE	20.3930	17.8625	17.8047	25.4575	18.1387	**17.7188**
	PSNR	21.9412	23.0920	23.1201	20.0145	23.1621	**23.1621**
*fish*	RMSE	18.4114	17.7298	17.5758	24.0806	17.3458	**17.2849**
	PSNR	22.8291	23.1567	23.2325	20.4975	23.3469	**23.3775**
*tool*	RMSE	8.5852	5.1631	5.2033	18.1161	4.8748	**4.8210**
	PSNR	29.4558	33.8726	33.8053	22.9695	34.3716	**34.4681**

### 4.2 Visual comparisons

**Fig 3:**
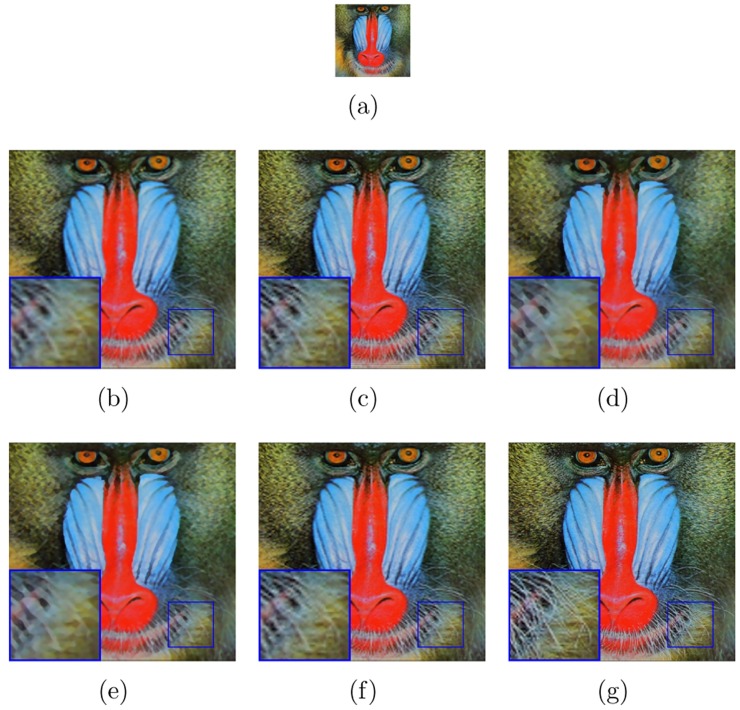
Results of “baboon” with the upscaling factor s = 3. (a) LR image, (b) bicubic, (c) 11’IPOL [2], (d) 14’TIP [10], (e) 10’TIP [12], (f) AHFM [7], (g) MAHFM.

**Fig 4:**
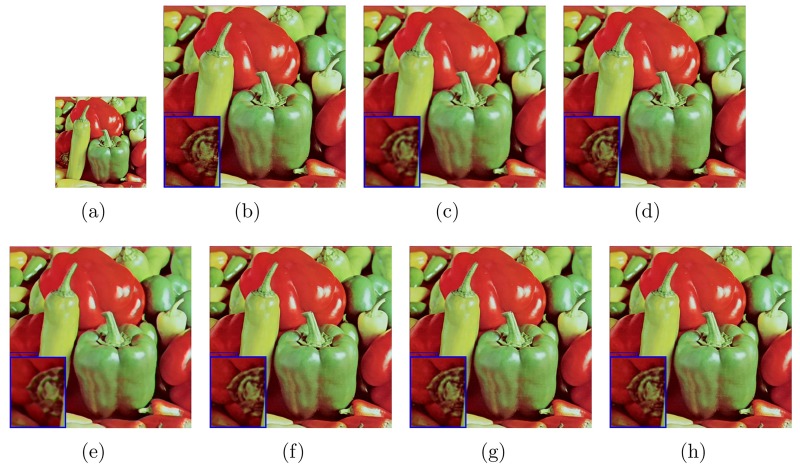
Results of “peppers” with the upscaling factor s = 2. (a) LR image, (b) ground truth, (c) bicubic, (d) 11’IPOL [2], (e) 14’TIP [10], (f)10’TIP [12], (g) AHFM [7], (h) MAHFM.

The test images (LR images) in Figs 3 and 4 are shown in Figs 3(a) and 4(a) of every figures, respectively. The image “baboon” and “peppers” are downloaded from http://decsai.ugr.es/cvg/dbimagenes/c512.php. The bottom of the web page has shown that these images are Copyright free. The version of the images in our paper are other special formats which are converted by Photoshop from the sources above. We have shown the results on these images with different upscaling factors. For example, we have set the upscaling factor *s* = 3 in the Fig 3. The proposed algorithm is compared with bicubic interpolation, a learning-based method (“10’TIP” [12]), one state-of-the-art interpolation-based methods (“11’IPOL” [2]), one deep learning method(“14’TIP” [10]) and the AHFM [7]. As can be seen, blur effect is generated by bicubic interpolation method. The learning-based method has comparable vision while we have to need extra training data to learn the relation between the test images and the training images. In addition, the interpolate-based methods (“11’IPOL”) also show impressive performance. In particular, “14’TIP” shows superior vision on image edges and runs quite fast. But as shown in figures, not only does it lose small-scale texture but also introduces minimal jagged artificial and smooths non-edge regions. By contrast, the recovered HR images by the MAHFM algorithm are preserved sharpness on the edges, such as the texture of the mustache without ringing and blurring artifacts. Compared with the ground truth image, the MAHFM alorithm generates superior effect than others not only visually but also quantitatively.

### 4.3 Comparisons between the AHFM and the proposed algorithm (MAHFM)

**Fig 5:**
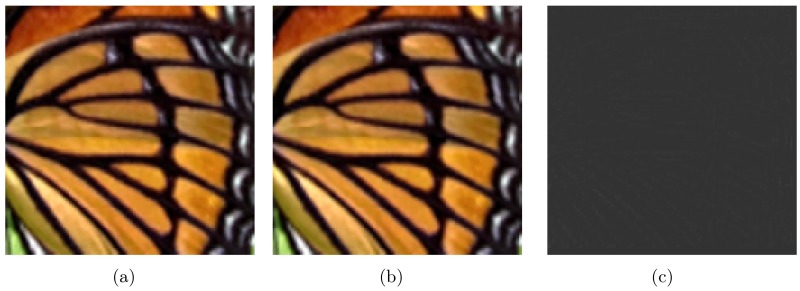
Comparisons between MAHFM and AHFM with factor *s* = 2. (a) MAHFM (RMSE = 8.7501, PSNR = 29.2905 dB). (b) AHFM [7] (RMSE = 9.0810, PSNR = 28.9681 dB). (c) residual (For better visualization, we add 0.5 to the intensities of the residual).

In this section we will illustrate the comparisons between the AHFM algorithm [7] and MAHFM algorithm. First, it is necessary to illustrate the difference between Algorithm 2 (let *l* = 1, *k* = 2) and AHFM algorithm. Although the only difference between the two methods is that the MAHFM algorithm is consisted of another term, which characters more sharp components. These could be seen much more clearly in Fig 5, which shows that the resulted HR image of Algorithm 2 (let *l* = 1, *k* = 2) is more sharp than that in the AHFM algorithm, especially at edges in Fig 5(c).

**Fig 6:**
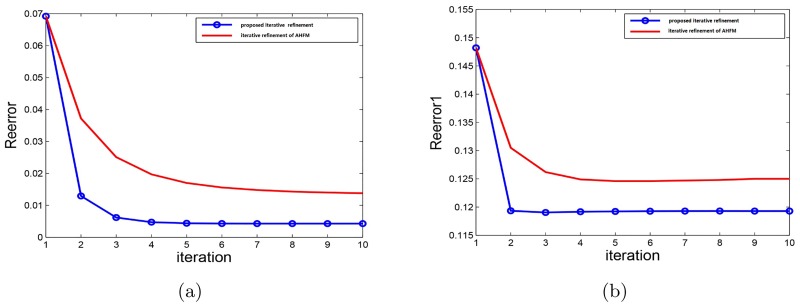
Comparisons between two iterative refinement methods. (a) Reerror. (b) Reerror1.

Alternatively, we also compare the performance of the iterative refinement generated with that of the iterative AHFM algorithm [7]. To measure the effect of iterative refinement, we employ two classes of relative error defined as following:

$$\begin{array}{c} \text{Reerror}=\frac{∥L-DH{∥}_{F}}{∥L{∥}_{F}}, \end{array}$$ (43)

and

$$\begin{array}{c} \text{Reerror1}=\frac{∥H_{true}-H{∥}_{F}}{∥H_{true}{∥}_{F}}. \end{array}$$ (44)

where *L* is a low resolution image. *D* is the downsampling operator. *H* is the last updated resulted image. *H*_*true*_ is the true high resolution image, and ∥ ⋅ ∥_*F*_ is the *Frobenius* norm. After computing the relative errors of all the test images, we take the average of all the relative errors as the final results. The final results compared with the iterative AHFM algorithm in [7] are plotted in Fig 6, illustrating the Algorithm 3 could produce more details and less error than the original iterative refinement method. Besides, the precision of the proposed method is higher than that in original method. From the Fig 6, we can also get the iterative steps of the iterative refinement method. Only two iterations could be achieved the goal of refinement, which would also reduce the operation time.

### 4.4 Parameters

**Fig 7:**
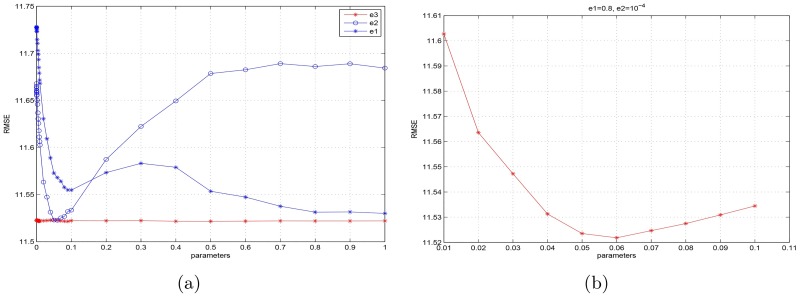
(a) The RMSE trend of the MAHFM algorithm as the different parameter varies when other parameters are roughly estimated (To simplify the experiment, *e*_1_, *e*_2_, *e*_3_ represents *ξ*_1_, *ξ*_2_, *ξ*_3_, respectively in this figure). (b) The RMSE trend of the MAHFM algorithm as the parameter *ξ*_2_ varies, where *ξ*_1_ = 0.8, *ξ*_3_ = 10^−4^.

As mentioned in section 3, there are many parameters in the MAHFM algorithm, e.g. *ξ*_1_, *ξ*_2_, *ξ*_3_, λ_1_, λ_2_, λ_3_ and others. Although the parameters are so many, they are easy to select. In our work, we select these parameters mainly according to the experience. In particular, the parameters *ξ*_1_, *ξ*_2_, *ξ*_3_ are especially important, because the sharpness for different components of an image are decided by them. First, we test *ξ*_1_ by fixing *ξ*_2_, *ξ*_3_ and then tune *ξ*_1_. In this way, we obtain a rough estimation of the three parameters (see Fig 7(a)). Note that there are some special points, which we can roughly estimate *ξ*_1_ ∈ [0.8, 1], *ξ*_2_ ∈ [10^−2^, 10^−1^] and *ξ*_3_ ∈ [10^−4^, 10^−2^]. Fig 7(a) as well as reveals *ξ*_1_ and *ξ*_3_ are not sensitive to the selection of parameters. Thus we can select *ξ*_1_ = 0.8 and *ξ*_3_ = 10^−4^ at first, and then tune *ξ*_2_ from 10^−2^ to 10^−1^ by 10^−2^ time change, which is plotted in Fig 7(b). We could find that *ξ*_2_ = 6 × 10^−2^ is the best fit. We conduct the same method to select other parameters. The final parameters of MAHFM algorithm are shown in Table 4.

**Table 4:** Parameters of MAHFM algorithm.

*ξ*_1_	*ξ*_2_	*ξ*_3_	λ_1_	*γ*_1_	*γ*_2_	*τ*_1_
1	6 × 10^−2^	1 × 10^−4^	5 × 10^−4^	1 × 10^−3^	1 × 10^−5^	3 × 10^−3^
*τ*_2_	${\tilde{\xi }}_{1}$	${\tilde{\xi }}_{2}$	*μ*_1_	*μ*_2_	${\tilde{\tau }}_{1}$	${\tilde{\tau }}_{2}$
1 × 10^−4^	1 × 10^−4^	1 × 10^−5^	1 × 10^−4^	5 × 10^−3^	1 × 10^−5^	1 × 10^−6^

### 4.5 Computation time

**Fig 8:**
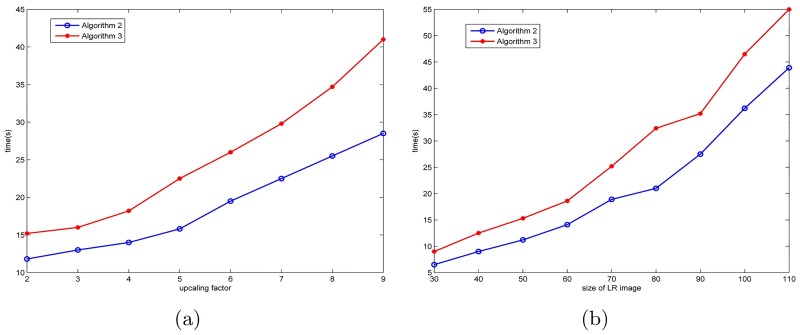
(a) Computation time of Algorithm 2 (*l* = 1, *k* = 2) and Algorithm 3 vs. upscaling factor for the low-resolution image “butterfly” with LR size 60 × 60. (b) Computation time of Algorithm 2 (*l* = 1, *k* = 2) and Algorithm 3 vs. the size of low-resolution image “butterfly”, the size of low-resolution image is increased from 30 × 30 to 110 × 110 and the upscaling factor is always set to be 4. Note that, to reduce the instability of Matlab, the computation time is the average of 10 runs.

**Fig 9:**
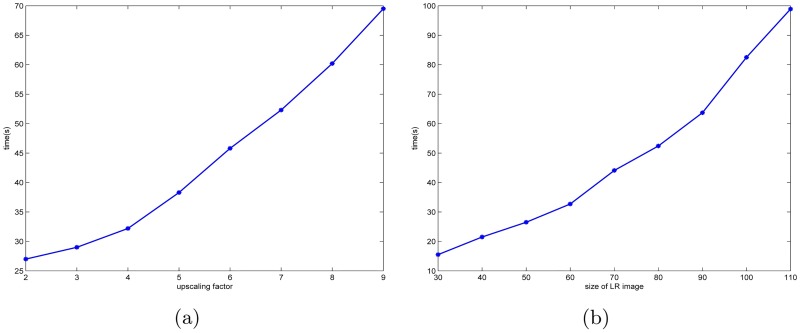
(a) Computation time of MAHFM (i.e., Algorithm 5) vs. upscaling factors for the low-resolution image “butterfly” with LR size 60 × 60. (b) Computation time of MAHFM vs. the size of low-resolution image “butterfly”, the size of low-resolution image is increased from 30 × 30 to 110 × 110 and the upscaling factor is always set to be 4. Note that, at the common point with Fig 8 (i.e., upscaling factor 4 and 60 × 60 LR size), it has slightly different computation due to the instability of Matlab, thus we present the computation time by the average of 10 runs to reduce the gap.

From Figs 8 and 9, we can see the proposed method is a little slower in operation time. The main reason is that our algorithm is mainly to compute the coefficients *u*, however, due to many components in the proposed algorithm have to be character thereafter there are a lot of loop programs used Matlab. Hereto, to speed up the computation time, there is a lot of room. We believe that the limitations could be improved by using cmex in Matlab involving a lot of loops to speed up the code. From Fig 8, we can also find that Algorithm 3 runs more time than Algorithm 2, since it involves more matrix-vector representation. In addition, in our work, we also use the theory of patch to speed up. Based on this, we can also consider utilizing the parallel computing.

### 4.6 The differences between Heaviside function and wavelets

**Fig 10:**
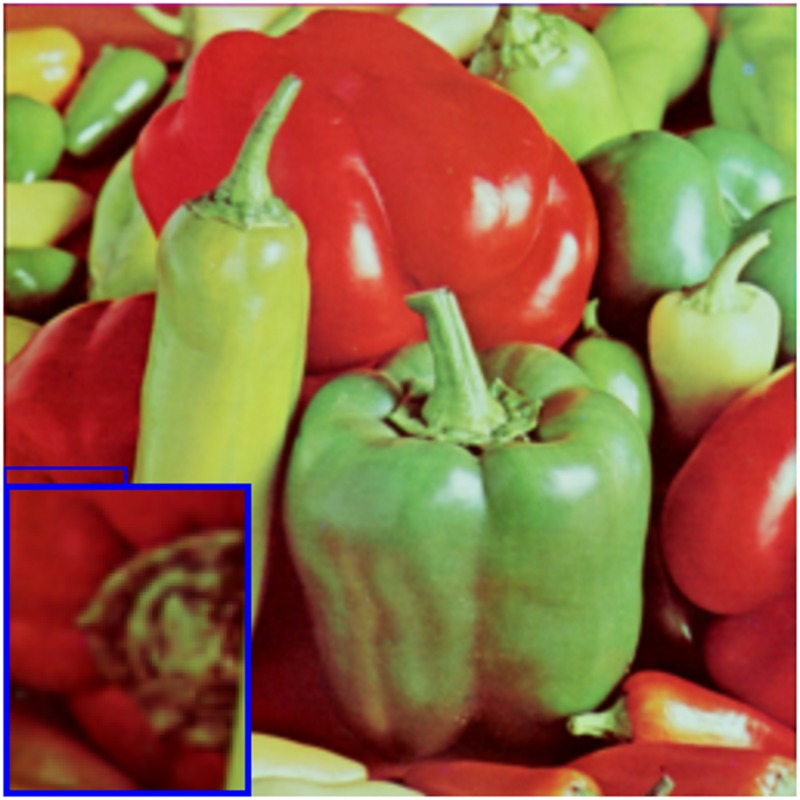
Results of “peppers” with the upscaling factor s = 2 via the representation of Haar wavelets.

In the image processing field, there exist some other basis functions such as wavelets [35]. Therefore, we discuss the difference between the Heaviside function and wavelets. To test the difference, we write a program about the representation of super resolution based on the haar wavelet. The quantitative comparisons are shown in Table 5. Compared Fig 10 with Fig 4, we can see that the algorithm based on Heaviside function has got more details about the non-smooth components than the wavelets do. The algorithm based on wavelets mainly splits one image into different sub bands. The first layer scale coefficients and wavelet coefficients of the image are extracted from the structure of wavelet decomposition. The different sub bands focus on the different ways, such as the information about horizontal, vertical and other directions. Every sub band persists the information only about the corresponding direction. If some operators, such as bicubic interpolation, are applied, error would be generated. And then the error would make pixels overlap, causing Gibbs effect. Differently, the basis is generated by the corresponding Heaviside functions and the representation coefficients are extracted from the low resolution image. The MAHFM algorithm could represent different sharp components via different classes of Heaviside functions as well as possible, which makes the information minimize the loss.

**Table 5:** Comparison with super resolution based on the basis of wavelets and Heaviside function.

Algorithms	RMSE/PSNR	*peppers* (*s* = 2)	*babyface* (*s* = 2)	*barbara* (*s* = 3)	*girl* (*s* = 3)	*baboon* (*s* = 3)
wavelets	RMSE	6.1932	5.1663	13.0254	6.2360	18.5015
	PSNR	32.2925	33.9518	25.8350	32.2327	22.7867
Heaviside function	RMSE	**4.6897**	**4.3713**	**11.8880**	**4.8210**	**17.7188**
	PSNR	**34.7080**	**35.3185**	**26.6286**	**34.4681**	**23.1621**

## Conclusion

In this paper, we have proposed a general framework of approximated Heaviside functions model that can describe different smooth components and non-smooth components in an image. The different components of an image could be approximately represented by different classes of approximated Heaviside functions (AHFs). With only one LR image input, we could compute the representation coefficients of AHFs, and then utilized these representation coefficients to obtain the high-resolution images. In addition, to pick up more image details, we employed an iterative refinement algorithm according to the residuals between the LR input and the downsampled LR image. To reduce computation and storage size, we applied the MAHFM algorithm to image patches. In particular, we discussed how to choose parameters. Extensive examples have been provided in the experiments to show the effectiveness of the MAHFM algorithm both quantitatively and visually.
